# Post-Traumatic Growth in Volunteers Following the 2023 Kahramanmaraş Earthquakes

**DOI:** 10.3390/ijerph22050699

**Published:** 2025-04-28

**Authors:** Kader Demiröz, Mehtap Kılıç, Sevda Demiröz Yıldırım

**Affiliations:** 1 Department of Emergency Aid and Disaster Management, Artvin Coruh University, 08000 Artvin, Türkiye; kader.demiroz@artvin.edu.tr; 2 Department of Emergency Aid and Disaster Management, Ardahan University, 75000 Ardahan, Türkiye; mehtapkilic@ardahan.edu.tr; 3 Department of Emergency Aid and Disaster Management, Burdur Mehmet Akif Ersoy University, 15100 Burdur, Türkiye

**Keywords:** disaster management, earthquake, post-traumatic growth, volunteer, Kahramanmaraş earthquakes

## Abstract

The 6 February 2023 Kahramanmaraş earthquakes were devastating events that caused widespread destruction. This mixed-methods study examined post-traumatic growth (PTG) in volunteers who participated in the relief efforts. A total of 169 volunteers participated in the quantitative phase, completing a standardized PTG measure. In-depth interviews were conducted with 14 volunteers during the qualitative phase. The study found that gender had a significant effect on total PTG scores. Additionally, gender, earthquake experience, and volunteer organization were significant factors in the “change in self-concept” sub-dimension. Gender was the only significant factor in the “change in philosophy of life” sub-dimension. Qualitative analysis revealed that participants experienced trauma symptoms after the earthquake but also reported positive changes in self-concept and life philosophy. This study suggests that disasters can lead to PTG, despite the presence of trauma symptoms. Further research is needed to explore PTG in different disaster response groups.

## 1. Introduction

Türkiye, situated in a seismically active region, is frequently exposed to various natural disasters. Earthquakes have historically constituted one of the most significant threats to the country. On 6 February 2023, a series of devastating earthquakes struck the Kahramanmaraş province, with magnitudes of Mw 7.7 (Pazarcık) and Mw 7.6 (Elbistan) at 04:17 and 13:24, respectively. These earthquakes significantly impacted 11 provinces, affecting an estimated 15 million residents [1]. The catastrophic events resulted in a staggering loss of life, with 50,783 fatalities and 107,204 injuries [2,3]. The widespread destruction encompassed over half a million buildings, and preliminary economic assessments indicate losses exceeding $34 billion USD [3]. Due to their magnitude and the extent of the devastation, these earthquakes are considered the most destructive in the nation’s history [1].

The overwhelming scale of the disaster rapidly overwhelmed local and national resources, necessitating international aid. Assistance initially flowed from neighboring provinces and subsequently occurred on a national level. A significant number of volunteers mobilized to the affected region, contributing to various relief efforts. Volunteers from diverse institutions and organizations actively participated in critical activities such as search and rescue, tent erection, and the sorting and distribution of essential supplies. While the overall number of volunteers remains uncertain, official records indicate 35,409 volunteers served under the auspices of AFAD and 48,411 under the Red Crescent [4,5]. The devastating earthquake centered in Kahramanmaraş had a profound and multifaceted impact, affecting not only the direct victims but also disaster responders, including officials and volunteers, and the entire nation. These events triggered widespread psychological trauma [6,7]. Trauma, characterized by overwhelming stress that exceeds an individual’s coping mechanisms, can arise from various sources, including death, accidents, illness, and disasters [8]. This phenomenon is a common human experience [9]. While trauma often leads to negative psychological, behavioral, and emotional consequences such as depression, anxiety, and post-traumatic stress disorder (PTSD) [10], some individuals demonstrate remarkable resilience and experience positive personal growth.

The evaluation of a stimulus by an individual, such as a disaster, and the subsequent emotional reactions that ensue, are subject to change [11]. These reactions are termed “emotion regulation”. It is an established approach that emotional intensity can be regulated through the implementation of specific strategies [12]. One such strategy is cognitive emotion regulation, which involves cognitive management and the evaluation of emotions, with the objective of resolving emotions that cause individuals distress through cognitive processes. In essence, cognitive emotion regulation strategies can be defined as cognitive coping mechanisms employed in response to warning events (e.g., accidents and disasters) [13]. Cognitive emotion regulation strategies consist of nine different strategies, which can be categorized as either compatible or incompatible. Whilst self-blame, the blame of others, rumination, and catastrophizing are considered to be maladaptive coping strategies, acceptance, refocusing on the plan, devaluing the event, and positive reappraisal are considered to be adaptive coping strategies [14]. Given the focus of this study on posttraumatic growth, it was deemed pertinent to examine the ‘positive reappraisal’ strategy in the context of the adaptive coping strategies of cognitive emotion regulation. Positive reappraisal, a positive coping strategy, is defined as the process of reinterpreting a negative situation in a positive light, thereby enhancing one’s sense of personal development in the face of adversity [15]. This process involves the cognitive reappraisal of the situation, where individuals adopt a positive perspective and interpret events in a way that is beneficial to their well-being [16]. Positive reappraisal has been defined as a cognitive process that involves the interpretation of a situation in a manner that is perceived to engender a positive emotional response in an individual confronted with a challenging circumstance [17]. Positive reappraisal has been shown to be indicative of thoughts that contribute to personal development, thereby creating positive meanings about the stimulus event experienced [18]. This strategy has been demonstrated to enhance psychological well-being and reduce anxiety [15]. Research has demonstrated that positive reappraisal is associated with a reduction in pain and depressive symptoms and a decrease in depression in the face of negative events [11,19,20].

Positive reappraisal has been identified as a coping strategy that is directly associated with the concept of posttraumatic growth. The posttraumatic growth (PTS) phenomenon has been observed in individuals who have experienced painful and stressful trauma. This phenomenon, known as post-traumatic growth (PTG), refers to the significant positive changes that individuals experience in response to the struggle with major life crises [9]. PTG encompasses personal transformation, such as increased self-awareness, a deepened appreciation for life, and a stronger sense of purpose. This concept is crucial for understanding societal resilience in the face of disasters. While professional disaster responders undergo rigorous training and preparedness exercises, volunteers may experience unique challenges and vulnerabilities. Therefore, examining PTG among volunteers is critical for enhancing disaster management strategies. Although research on PTG has been conducted across various domains, studies specifically focusing on volunteers involved in the 2023 Kahramanmaraş earthquakes are currently lacking. This study aims to fill this gap by investigating the levels of PTG among individuals who served as volunteers in the affected region.

## 2. Materials and Methods

The present study employed a mixed-method approach as its research method. In mixed-method research, quantitative and qualitative data are collected and analyzed to address research questions. The strengths of both qualitative and quantitative methods are utilized, and these two research methods are combined and integrated through special mixed-method designs. Consequently, the researcher combines statistical trends with narratives and personal experiences to enhance the comprehension of the research problem [21]. In this study, the exploratory sequential mixed-method approach was employed, which is considered a fundamental mixed design. This approach involves the initial collection and analysis of quantitative data, followed by the formulation of subsequent stages based on the findings from the initial analysis. The second stage involves the collection and analysis of qualitative data. The amalgamation and interpretation of all collected data enables a comprehensive investigation of the research question.

Purposive sampling was employed as the method for selecting the sample. The inclusion of individuals with specific characteristics in the sample group is considered purposeful sampling [22,23]. The participants in this study were volunteers who went to the field during the 6 February 2023 Kahramanmaraş earthquakes. In this context, the inclusion criteria were as follows: to have worked as a volunteer in the regions devastated by the Kahramanmaraş earthquake, to have worked in face-to-face dialogue with the disaster victims, to be over 18 years old, and to have participated in the research voluntarily. People who did not meet all the inclusion criteria were not included in the research.

In this context, individuals with the relevant characteristics were included in the study. The selection of volunteers was predicated on the prediction that individuals lacking experience in disasters and analogous crisis situations would be disproportionately impacted. Consequently, this study was devised to assess the PTG levels of volunteers. The sample size for the quantitative study was determined using the web-based OpenEpi website [24]. It was reported that 83,820 volunteers were deployed under the Red Crescent and AFAD organizations. It is acknowledged that there are volunteers who went to the field but were not recorded. However, the exact number is not clear, but after a certain number, the sample number is stated as 165 at an 80% reliability level. The number of datasets constituting the quantitative part of the study is 169, which meets the 80% reliability requirement. The qualitative component of the study involved 14 participants, and the interviews were concluded when data saturation was reached. A total of 426 min of interviews were conducted with these 14 participants.

The “Posttraumatic Growth Inventory” (PTGI) was utilized to gather quantitative data in the present study. The PTGI was developed to ascertain the perspectives of individuals who have encountered traumatic events in their life post-trauma. This scale was conceptualized by Tedeschi and Calhoun [25] and encompasses three dimensions and twenty-one items: “Change in Self-Concept”, “Change in Philosophy of Life”, and “Change in Relationships”. Each item is graded between 0 (I did not experience this change at all as a result of the stressful event(s)) and 5 (I experienced this change to a great extent as a result of the stressful event(s)). In this context, the total score that can be obtained from the scale varies between 0 and 105. The score ranges according to the sub-dimensions were determined as 0–50 for Change in Self-Concept (10 items), 0–30 for Change in Philosophy of Life (6 items), and 0–25 for Change in Relationships with Others (5 items). High scores indicate that the individual experienced more positive psychological development in the posttraumatic process and that there were significant transformations in self-perception, the understanding of life, and interpersonal relationships. Low scores, on the other hand, indicate that posttraumatic development was limited or not realized. This scoring system makes it possible to interpret the findings obtained in the study in a more accurate and meaningful way.

Parametric test assumptions were evaluated in the analyses related to PTGI total score and sub-dimensions. The normality assumption was tested with the Kolmogorov–Smirnov test since the sample size was over 50. Values of *p* > 0.05 were obtained for the PTGI total score and “Change in Philosophy of Life” sub-dimension. Although *p* < 0.05 was observed in some groups in other sub-dimensions, it was accepted that the data were normally distributed because the skewness and kurtosis values were within ±1 limits and the sample size was sufficient (*n* > 30). Therefore, parametric tests such as the t-test and ANOVA were found to be appropriate.

To collect qualitative data, researchers developed a semi-structured interview form. This interview form was designed to assess the PTG of volunteers who played an active role in the disaster response process following the disaster. Initially, a set of draft questions was formulated, and the semi-structured interview form was finalized based on the analysis of quantitative data. The initial part of the form comprises demographic inquiries designed to facilitate the interviewer’s familiarization with the subject, while the subsequent section delves into PTG levels, aligning with the PTGI.

In order to ensure the internal validity of the study’s validity criteria, long-term interaction, confirmation, strategies to reduce researcher bias, and researcher triangulation technique were used, while for external validity, steps such as purposive sampling selection, the determination of selection criteria, and environment and participant introduction were followed [26,27]. The following steps were taken to enhance the validity and reliability of the study: To ensure internal validity, the interview form was developed based on a thorough review of the relevant literature. Additionally, to ensure external validity, the interviewees were informed about the research processes, research purpose, research model, data collection method, and data analysis. To ensure internal validity, the findings were presented directly without interpretation, analyzed by three independent researchers, and the thematic appropriateness was reviewed. The themes and codes that emerged from this analysis were then reviewed by two researchers specializing in qualitative research, who offered their insights and suggestions.

In the study by Kağan et al. (2012), the validity and reliability of the Turkish version of the PTGI were thoroughly evaluated [28]. Utilizing structural equation modeling, confirmatory factor analysis revealed the three-factor structure of the scale to be valid. These three factors were identified as Change in Self-Concept (CSC), Change in Philosophy of Life (CPL), and Change in Relationships with Others (CRO). The internal consistency of the scale was found to be high as α = 0.92 for all items, and the internal consistency coefficients of the subscales ranged between 0.77 and 0.88. In addition, 15-day test–retest reliability coefficients were 0.83 for total scores and 0.70 to 0.85 for subscales. These findings underscore the validity and reliability of the PTGI (Post-Traumatic Growth Inventory) as a measurement instrument in the Turkish context, thereby providing a robust quantitative foundation for the study’s implementation.

Research Hypotheses and Research Questions

Hypotheses

***H1:***  *Gender exerts a significant influence on PTG levels and sub-dimensions.*

***H2:***  *Age groups demonstrate significant variations in PTG levels and sub-dimensions.*

***H3:***  *Educational level exerts a significant influence on PTG levels and sub-dimensions.*

***H4:***  *Marital status demonstrates a significant impact on PTG levels and sub-dimensions.*

***H5:***  *Region of residence shows a significant effect on PTG levels and sub-dimensions.*

***H6:***  *Experiencing an earthquake has a significant impact on PTG levels and sub-dimensions.*

***H7:***  *The organization in which the volunteering activity is carried out exerts a considerable influence on the levels and sub-dimensions of PTG.*

The following research question was posed:

How do volunteers who participated in relief efforts following the 2023 Kahramanmaraş Earthquake interpret the experience, and how do they define PTG related to this process and its aftermath?

Ethical Considerations

Prior to the initiation of the study, ethical approval was obtained from the *** (Number: ***). Separate study participation approval was obtained for each form during the data collection process. Moreover, audio and written permission was obtained from the participants in the qualitative data collection process for audio recording before the interview. The protection of personal data was prioritized throughout the study, and participants were informed that the utmost care was taken to safeguard their information.

## 3. Results

### 3.1. Quantitative Results

The relevant information in line with the analyses made within the scope of the scales and forms prepared to determine the PTG Level is available in Table 1.

As indicated in Table 1, 46.7% of the participants identified as female, while 53.3% identified as male. With respect to age, 56.2% of the participants fell within the 18–25 age range, 29.6% were between 26 and 35 years old, and 14.2% were between 36 and 45 years old. Although this situation is outside the inclusion criteria, it shows that the average age of volunteers is low and the volunteer population is young. In Türkiye, the age of participation for disaster coordination volunteers is 15 years for AFAD volunteers and 18 years for AFAD support volunteers (AFAD volunteers who can participate in search and rescue operations). In order to ensure internal validity in the validity criteria of the study, long-term interaction, confirmation, strategies to reduce researcher bias, and researcher triangulation technique were used, while for external validity, steps such as purposive sampling, the determination of selection criteria, environment, and the introduction of participants were followed [29]. Concerning their educational attainment, 14.2% of the participants had not obtained a bachelor’s degree, 75.1% had obtained a bachelor’s degree, and 10.7% had obtained a postgraduate degree. With respect to marital status, 23.1% of the participants were married and 76.9% were single. In terms of geographical distribution, 63.3% of the participants resided in the province, 32.5% in the district, and 4.1% in the village. It is noteworthy that 67.5% of the participants had previously experienced an earthquake, while the remaining 32.5% had never encountered one. In terms of the institutions within which the participants engaged in volunteering activities in the disaster area, 37.3% of them participated in volunteering activities under AFAD, 7.7% under the Red Crescent, 21.3% under other non-governmental organizations, 18.3% under the umbrella of the public institutions or private sector organizations they work for, and 15.4% under their own means.

A comprehensive evaluation of the PTG levels of the volunteers was conducted, encompassing the sub-dimensions of the scale and various variables. Table 2 presents the sub-dimensions and total scores of the PTGI, categorized according to the sociodemographic characteristics of individuals engaged in various volunteering activities in the field during the Kahramanmaraş earthquake.

According to Table 2, the PTG of people who participated in volunteer activities in the earthquake show a significant difference according to gender, t (167) = 2.66, *p* < 0.05. Women (=3.90) who participated in volunteer activities have higher levels of PTG compared to men (=3.49). According to this finding, it can be said that there is a significant relationship between PTG level and gender.

When the sub-dimensions of the scale are analyzed according to all socio-demographic variables in Table 2, the sub-dimension of CSC (t (166.34) = 2.80, *p* < 0.05) and the sub-dimension of CPL (t (167) = 2.72, *p* < 0.05) show a significant difference according to gender. The sub-dimension of CSC shows a significant difference according to the status of experiencing an earthquake (t (167) = 2.007, *p* < 0.05). The sub-dimension of CSC shows a significant difference according to the institution in which individuals participated in volunteer activities (f (2, 164) = 2.555, *p* < 0.05).

However, in order to test whether this difference was affected by demographic variables such as gender, an analysis of covariance was conducted by controlling gender. In this way, the independent effects of the earthquake experience and the organization in which the volunteering activity was participated on posttraumatic development were evaluated. According to the analysis of covariance controlling for gender, a statistically significant difference was found between earthquake experience and CSC scores. This result reveals that even after the effect of gender is removed, individuals with earthquake experience show higher posttraumatic development in the dimension of self-perception. In addition, the assumption of homogeneity of variance was tested with Levene’s test (F (1, 167) = 3.40, *p* = 0.067) and found to be met. According to the analysis of covariance controlling for gender and earthquake experience, a significant difference was found in CSC scores according to the volunteer organization (F (4, 162) = 3.24, *p* = 0.014, η^2^ = 0.074). In the same analysis, the effect of gender (F (1, 162) = 9.66, *p* = 0.002) and the effect of earthquake experience (F (1, 162) = 6.89, *p* = 0.010) were also statistically significant. The model was significant overall (F (6, 162) = 4.43, *p* < 0.001) and explained 14% of the total variance (R^2^ = 0.141). These findings indicate that the effect of the volunteer organization on CSC is independent of other variables.

### 3.2. Qualitative Results

The demographic information of the volunteers who participated in the qualitative research and information about the duration of the interviews are given in Table 3.

#### 3.2.1. Theme 1: Roles in the Field

Participants were engaged in various field activities, including logistics, the identification of disadvantaged groups, shelter, coordination, and health services. These participants subsequently provided detailed descriptions of the processes associated with these activities.

*I can say that I was very helpful with logistics. After Islahiye, I moved to Pazarcık. I worked in Pazarcık X Tent City, but our only big problem was that we didn’t have a tent for logistics. The first time I went there, I saw that the materials were stacked in four different tents, which really bothered me. As soon as I arrived, I sorted the tents and materials into four categories: hygiene, clothing, food, and technical materials. We also had warming tents. I coordinated this area with my team. I was responsible for logistics*

(P1).

*My job is disaster management, so I worked on many issues in the tent city area. I had worked with AFAD representatives before, so they knew me. I had taught them disaster training on various subjects, so they asked me about many issues. I did many things, like sorting, classifying, and distributing incoming aid; working with other necessary institutions to keep the tent city safe; figuring out and fixing the needs of disadvantaged groups; asking the tent city for what it needed and following up on those requests; providing psychosocial support; receiving incoming aid; and organizing aid from non-governmental organizations in the tent city. In short, I worked on almost every issue in the tent city*

(P12).

*I can say that we provided continuous support in setting up the tents, especially with the support of the soldiers. We placed people in tents in line with the disaster triage. We participated in search and rescue activities in case of need. We distributed food, water, wood, and cleaned the area. We had a pharmacy and health services, and we provided first aid activities*

(P2).

*I was in charge of managing social media to get notifications and deliver them to the teams. I did this for 10 provinces. People sent me reports from their social media accounts, which I delivered to the teams, including the provinces, one by one via WhatsApp groups. I also coordinated the vehicles, planes, etc. of the teams coming from other provinces, assigned provinces, etc. At the same time, I was sharing the news that came to us on social media accounts, such as this person was taken alive in this apartment or that area and delivering them to people. This was actually my active duty*

(P14).

*I worked on the health services provided in field hospitals, and then on logistics. Now, field hospitals were established in our region from other countries. One of these was a field hospital established by the Americans. These countries returned to their own countries without field hospitals, and many of these field hospitals were given to certain regions so that they could be used in case of a possible disaster. After the Americans returned to their own countries, we provided logistical support to the ongoing health activities there*

(P6).

#### 3.2.2. Theme 2: Emotions

The participants were asked questions about how they felt while they were in the field and after they returned home. The participants used expressions that showed they felt bad both in the field and after returning from the field. While they felt helpless and sad while they were there, they felt most guilty after they returned.

##### Emotions in the Field

**Figure 1:**
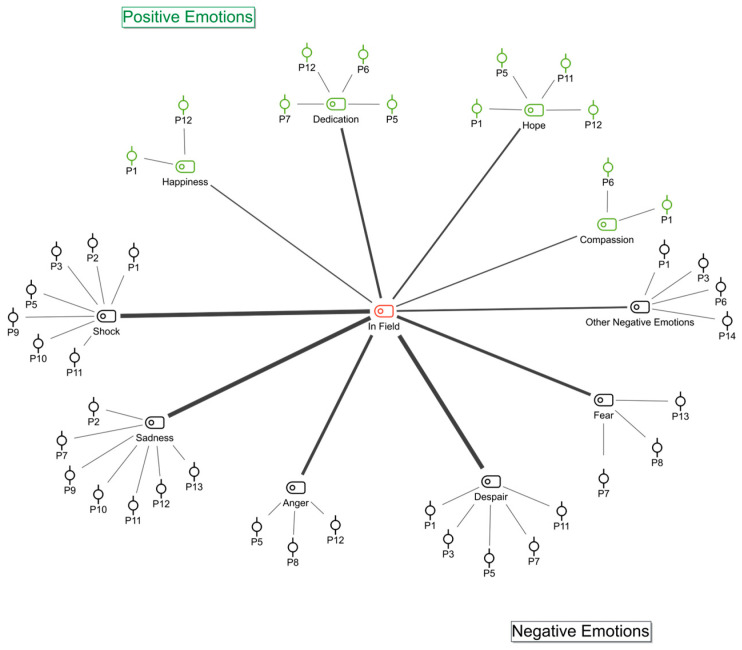
Participants’ emotions in the field: code–subcode–section model. Black shapes represent negative emotions and green shapes represent positive emotions.

The participants were asked questions about their emotions during their time in the field. These emotions were grouped into positive and negative categories (Figure 1).

Participants said that positive emotions in the field were compassion and self-sacrifice. They also said they felt hopeful and happy. They said this about positive emotions:

*When we saw the devastation, of course, we first felt sadness, but I think that the sense of compassion of the other personnel, like me, was at the highest level when we were already involved in these organizations. I felt that compassion was overwhelming, and I wanted to help the people affected by the disaster as much as possible*

(P6).

*…I was hopeful and happy because our citizens were sensitive and kind. While this was happening, I felt many different emotions at once*

(P12).

The most frequently mentioned negative emotions experienced in the field by participants were sadness, shock, and helplessness. In addition, fear and anger were among the other emotions that were coded. It was determined that the most frequently coded emotions were helplessness and sadness.

*I saw helplessness. I’ve always thought of myself as someone who knows himself very well and never sees himself as helpless. I always believe there is a way out, and that’s the quality I admire most about myself. I’ve never said, ‘Alright, the road ends here, this is it’. I always look for solutions. But when I saw the state of those people there, I realized that helplessness truly exists. People can become helpless. The father of the child we rescued from the rubble came to me. He said, “I found my child. I go to him, touch him, kiss him, love him. He says, ‘Dad, save me,’ but there is nothing I can do. “That is helplessness. There is nothing beyond that. I’m a father too, and I can’t forget this. I truly understood what helplessness is, down to my very core, from the state and condition of those people there*

(P3).

*I was shocked to see my hometown in such bad shape. I love Hatay so much, and it was heartbreaking to see the city in such poor condition. I couldn’t believe it, and it felt surreal*

(P10).

*I was extremely scared and worried. I’ve never been so scared in my life, nor have I ever faced death so closely. Imagine, at that moment (due to the earthquake), everyone was running in a certain direction. It was the feeling that something terrible was happening, and I thought to myself, ‘We must have come here to die’*

(P8).

*My undergraduate education was in disaster management, so it affected me even more. We’ve heard these terms and concepts so many times in our lives, from past earthquakes. We also studied disaster management in our courses. I was directly involved in disasters. I saw helplessness, children standing barefoot outside trembling in those harsh winter conditions, and people in utter despair. This pushed me to a feeling of anger*

(P5).

*To be honest, while I was working, I was really angry. I saw a lot of destruction, and it could have been avoided. I also remember the children, women, and elderly people crying. I will always remember what they went through*

(P12).

*I learned a lot about myself there. I realized that I’m not the type to cry easily, get angry quickly, shout, or stay calm in crisis situations. Instead, I found myself laughing a lot as a way to balance everything. Of course, this might also be due to hormonal responses or other factors. I think it was a way for my brain to cope, and it helped me relax and save energy for the future*

(P2).

##### Subsequent to Returning from the Field

**Figure 2:**
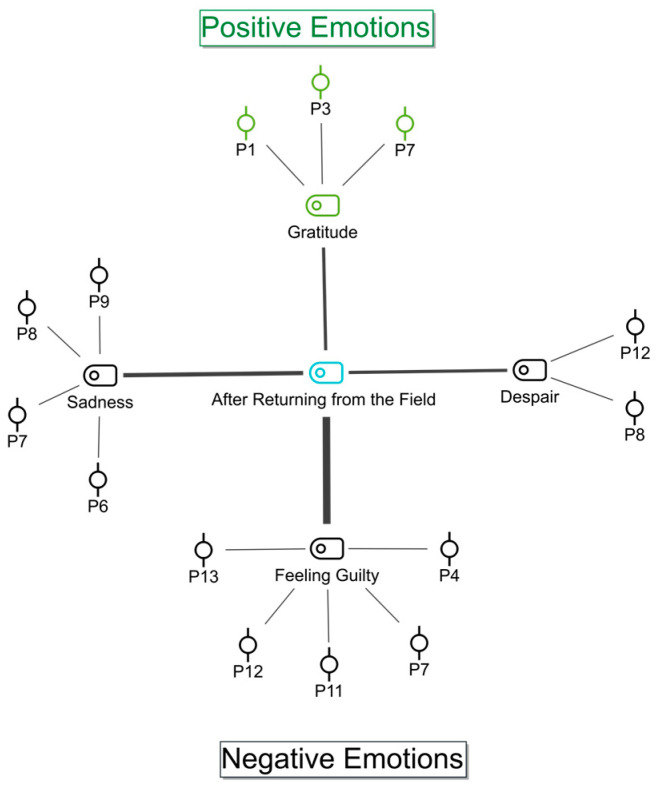
Participants’ emotions subsequent to returning from the field: code–subcode–section model. Black shapes represent negative emotions and green shapes represent positive emotions.

The participants were queried about their emotional state during the post-field process, and these emotions were classified as positive and negative emotions (Figure 2).

It was ascertained that the most frequently coded emotion was a sense of guilt. The participants stated that, as a positive emotion, they only experienced a feeling of gratitude after returning from the field.

*I realized that I had a lot in my life. I was still healthy. I had a warm home and a comfortable bed to sleep in. In fact, I had a comfortable life. Maybe what wouldn’t be considered comfortable for many people started to feel extremely luxurious to me. So, in general, I was feeling grateful. My family was alive, and I was healthy*

(P7).

It was explained by the participants that, upon their return from the field, they experienced a range of negative emotions, including feelings of guilt, sadness, and a sense of hopelessness.

*I felt guilty because there was no life left there, but when I returned, I had a home, a life, and the chance to go outside. Over there, people don’t even have workplaces anymore. They don’t even have jobs to go to. Routine is actually such a precious thing*

(P11).

*After returning from the field, I isolated myself from people quite a bit. I especially stayed away from people who acted like nothing had happened, like people hadn’t died there, or who spread false and misleading information to manipulate others. This saddened me*

(P9).

*I feel hopeless because even if you have a lot of knowledge, you can’t do anything if you don’t have the authority. You can’t say what you want to say, and everything gets stuck in your throat. I have my silence and my silent screams because when a person can’t do what they’re capable of, I think they freeze at some point. That’s why I was silent; I didn’t even want to talk to anyone about it. I did receive invitations afterward, but I didn’t want to do anything. During that period, I didn’t even want to talk about the incident because there was so much to say, but I felt, “Who should I say it to?” I experienced that kind of exhaustion*

(P8).

#### 3.2.3. Theme 3: Severe Trauma Symptoms

An analysis of the data revealed findings indicating that volunteer individuals exhibited severe trauma symptoms due to the earthquake, including sleep disturbances (most frequently), self-isolation, and the sensation of reliving the earthquake. The participants described their experiences regarding this issue as follows:

*I started having very bad dreams. I was so tired that I really needed to sleep. But I would wake up many times during the night. I could fall back asleep right away, but I kept waking up. This was the biggest physical problem I faced. The dreams were so intense and heavy that they really affected me. They even caused nightmares that felt like trauma. This is still happening*

(P2).

*I came back, but I really didn’t want to. My social life was really affected at first, including my relationships with other people. I didn’t even want to talk because whoever I saw would ask me about that moment. But for me, it was a really traumatic experience, and I didn’t want to be asked about it. Yet they kept insisting, asking questions. I would answer, and they’d keep asking. I’d say, “How was it? What could the situation be like?” I mean, eleven cities were affected. Everything was in ruins; how else could it be? For a long time, I talked to people with a very sullen attitude. Honestly, how could I smile when I knew a ten-day-old baby was lying in a tent in minus twelve, minus thirteen degrees?*

(P1).

*After returning from the field, I feel like I isolated myself. I distanced myself from my surroundings and people. Apart from my immediate family and close relatives, I somewhat withdrew from others*

(P10).

*“I often feel like there’s an earthquake happening all the time. While I used to be calmer and more composed during earthquakes, now, when they occur occasionally in the area where I live, my responses have started to differ from what they used to be. In a way, this is a good thing, of course. For example, I immediately take the drop, cover, and hold position or quickly try to choose a safe area. These are positive actions. But because I act so quickly, I’m less calm now*

(P2).

#### 3.2.4. Theme 4: Post-Traumatic Growth

**Figure 3:**
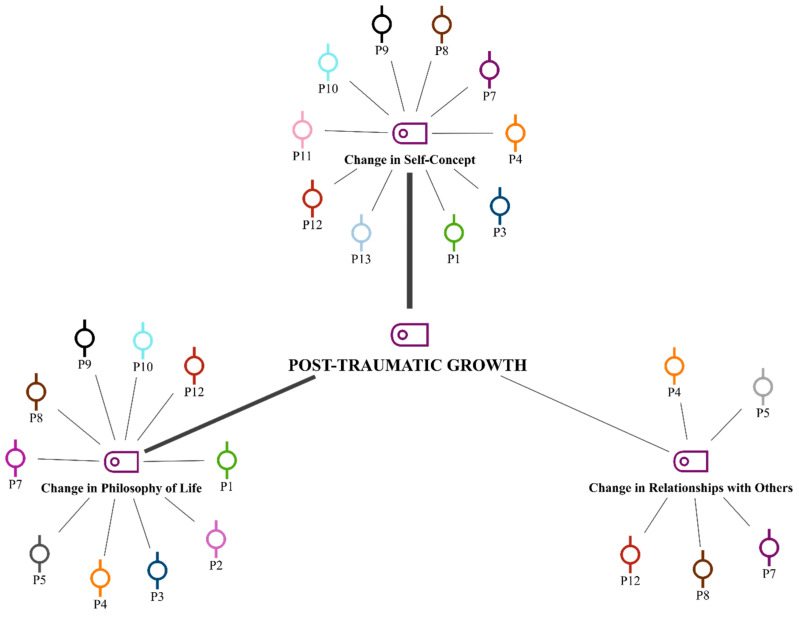
Theoretical model of PTG levels: codes and sub-codes. The line thicknesses in the figure show the frequency density.

To assess levels of PTG, participants were asked: “How would you compare your attitudes, thoughts, and perceptions of yourself and your surroundings before the earthquake to those after returning from the field?” Responses were analyzed and coded based on the relevant dimensions of the PTG scale. The analysis indicated that the most frequently observed change within the dimensions of PTG was in self-concept. This was followed by CPL and interpersonal relationships, respectively (Figure 3).

Participants were invited to elaborate on their experiences regarding CSC. The following is a summary of their responses:

*Honestly, my life changed a lot after the earthquake. You could say it was a turning point. I started being more intentional about how I direct my life and became more understanding and patient when talking to the people in my life. Because the person I hurt today could be the one whose funeral I attend tomorrow morning*

(P9).

*Don’t try to care about anything. I mean, most of the time, the things I used to care about or stress over, I now think, ‘This isn’t really a big deal’, or ‘Life is too short for this’. That kind of mindset developed*

(P10).

*I’m an intensive care nurse. Solving problems is routine for me. Something needs to be treated, it’s dealt with, the rules are clear. I was like that when I went there, too, but after spending time there and seeing what I saw, I realized that life doesn’t work like that. It actually taught me to accept what happens to me. Before going there, everything in life was something to fight for. But after returning, I learned that some things can’t be fought against*

(P11).

*I also realized more clearly that the most valuable things in life are time, health, and kindness. I started to value myself much more as well*

(P12).

*I became more understanding and learned that I can’t control everything. Before, I used to want to control everything and everyone. Now, after returning, I know that I can’t, and I can say that I’ve become calmer and more understanding*

(P13).

The participants shared their reflections on the shifts they had undergone in their life philosophies, articulating their insights as follows:

*The earthquake and the situations I had to face afterward should have left me depressed for months. For me, it would have been not getting out of bed, cutting off ties with my social circle, crying constantly, and being in a state of despair. But it reached a point where now, even if I find myself on the verge of depression over something, I stop and say, ‘What’s going on? Don’t be ridiculous! This isn’t worth it.’ That’s how I’ve changed*

(P4).

*For both me and those around me—for the public and the community—the only point I really focus on now is ethics. How can I work more ethically? How can this management be more honest? How can the people around me behave more ethically? We need to act ethically. I constantly have this question in my mind. Even in the smallest action I take, the smallest behavior, I now think, ‘How can I act more ethically? How can I act more fairly and honestly?’ This mindset has taken root in me after the earthquake, compared to before it*

(P5).

Participants articulated their perspectives on changes in their relationships with others as follows:

*My family bonds were already strong, but after the earthquake, they became even stronger. Before the earthquake, it was normal for me not to talk to them every day; talking once every two days wasn’t an issue. But after the earthquake, many things changed. Especially my family relationships became much stronger*

(P7).

*I already live alone, but at that time, the school was reopening, and students had returned. I started to become more sensitive and attentive toward my students, my family, and other people. I constantly called to ask, ‘How are you? Are you okay? Do you need anything?’ My sense of connection grew even stronger. For my students, I would like to ask, ‘Do you need anything? Is there anything I can do for you? Do you have a place to stay?’ I even invited some to my home, thinking they might not have a place to stay. I said, ‘You can come and cook whatever you like, you can use my place.’ I became much more helpful. I also had this constant urge to call my family. I didn’t feel very alone because, during that period, I was always in touch with my friends and family. Even though we weren’t physically together, we were constantly communicating over the phone*

(P8).

### 3.3. Qualitative Results Obtained from the Synthesis of Quantitative and Qualitative Data

As part of the study, quantitative analyses were conducted using the PTGI to determine whether the volunteers who participated in the field exhibited PTG and, if so, which subdimension was most prominent. The analysis revealed that the volunteers’ levels of PTG were above average, with the most significant change observed in the dimension of self-concept.

The standard deviation, median, and dispersion values presented in Table 4 were obtained from the PTGI scores of 169 volunteer participants who participated in the quantitative dimension of the study.

The mean PTGI total score of the participants ($\bar{x}$ = 3.71) is higher than the mean score per item ($\bar{x}$ ≈ 3.12) obtained by Kağan et al. (2012) [28] in the normative sample. In that norm study, the total score for 21 items was reported as 65.55, which corresponds to approximately 3.12 when divided by the number of items. In addition, the fact that the mean scores were higher in the “CSC” sub-dimension compared to the other dimensions in this study and significant differences were found in this dimension according to gender, earthquake experience, and the organization where the volunteerism was carried out reveals that this dimension is the dimension with the most significant change.

Table 5 is a summary of the questions prepared within the scope of the posttraumatic growth inventory and the coding performed in line with these questions. As a result of the analysis of qualitative data in order to detail the quantitative analysis and to collect in-depth data on the subject, it is seen that the dimensions of change in self-perception and change in life philosophy (*n*: 10) were coded intensively in terms of the number of people, but the coding for change in self-perception was higher than the other dimensions. Therefore, it can be said that a change in self-perception is perceived by the participants more than other levels of posttraumatic growth. In both sections (qualitative and quantitative), change in relationships with others was emphasized at the lowest level compared to other sub-dimensions. Therefore, within the scope of the analyses conducted in this study, it can be concluded that CSC among the volunteers was more pronounced compared to other dimensions.

## 4. Discussion

This study examined the PTG levels of individuals who participated in volunteer activities under the umbrella of various institutions and organizations during the Kahramanmaraş earthquakes. According to the research findings, PTG levels exhibited a significant difference only with respect to the gender variable. Additionally, the subdimension “CSC”, one of the subdimensions of the scale, showed significant differences not only by gender but also based on earthquake experience and the type of institution where the individual volunteered. Furthermore, the subdimension “CPL” was found to differ significantly only with respect to the gender variable.

Research indicates that individuals who derive meaning from their post-disaster experiences and achieve personal growth during this process are at a lower risk of experiencing mental health problems. This phenomenon is referred to in the literature as “post-traumatic growth” [30]. Various studies emphasize that PTG may vary depending on individuals’ socio-demographic characteristics. In this context, variables such as age, gender, marital status, educational level [31,32], previous trauma history, number of tasks performed, and coping styles have been examined in detail for their effects on PTG [31].

It is frequently reported that women exhibit higher PTG levels compared to men. In the literature, there are studies that demonstrate a significant relationship between PTG and gender, as well as studies that do not support this relationship [33]. Some research suggests that gender differences have positive effects on PTG [34]. Women’s tendencies to process emotional experiences more deeply and actively seek social support are considered facilitators of PTG [25,35]. Additionally, cultural and societal gender roles influence how individuals make sense of traumatic experiences and respond to them, thereby shaping PTG processes [36]. In this context, interventions aimed at promoting PTG should consider gender differences to better support individuals’ recovery processes. Moreover, our findings indicate that individuals who experience PTG are less likely to develop psychological problems in the long term and hold the potential to enhance the consistency of research in this field [33].

Experiencing a traumatic event emerges as a significant variable influencing individuals’ levels of PTG [37]. However, this relationship must be examined in the context of factors such as the time elapsed since the trauma, the severity of the trauma, and the level of individual exposure. In a study conducted by Yılmaz and Şahin (2007) on search and rescue workers, predictors of growth included previous trauma experiences, education level, frequency of participation in missions, positive emotions related to the job, basic assumptions, and general symptoms [31]. According to the findings of our study, the “CSC” subdimension of the PTG scale significantly varied depending on the earthquake experience of the volunteers. Additionally, significant differences were identified among the institutions where the individuals conducted their volunteer activities. These differences were attributed to the training provided by the institutions, which positively impacted PTG levels. It is assumed that such training transforms individuals’ concepts of traumatic experiences and supports personal growth during the process.

Voluntary mobilization by citizens following disasters is a common societal characteristic [38,39]. Volunteer processes are becoming increasingly significant for their contributions to societal recovery and disaster crisis management [40,41]. As such, support for volunteers to assume more prominent roles in disaster response processes across various tasks is steadily increasing [42,43]. Volunteers participate in various phases of risk and crisis management in disaster management and support disaster personnel. Volunteer activities in disaster management include disaster awareness campaigns, drills, search and rescue, medical support, equipment supply, debris removal, tent city setup, and the distribution of disaster aid [44]. It was determined that the volunteers in this study, like those in the literature, carried out activities in logistics, identifying disadvantaged groups, shelter provision, and coordination in disaster areas. Volunteers were found to provide support both in areas requested by public administration and in areas where they had expertise. Contributions from volunteers with expertise and prior experience in disaster management positively impact the success of disaster management activities. Furthermore, volunteers are more likely to participate in aid efforts within their areas of expertise [45]. Therefore, as indicated in the participants’ statements, effectively utilizing the expertise of volunteers enables a diverse range of volunteer activities to be successfully carried out during disasters.

During their time in disaster areas, participants reported experiencing positive emotions such as compassion, self-sacrifice, hope, and happiness. After returning from the field, participants indicated increased awareness and gratitude for their lives and existence due to the values they held. Positive emotions experienced in disaster areas were found to stem from two main factors: the sense of helping others, or being useful, and the strength of social capital. Volunteers demonstrated compassion and self-sacrifice in their efforts to help those in need, and they experienced hope and happiness due to the emphasis on societal solidarity in disaster areas. Studies conducted worldwide indicate that helping and serving the community are primary motivations for volunteering [46]. Social capital, defined as features that enhance the community’s productivity, trust, and communication networks [47], not only increases the capacity to cope with disasters but also benefits from the presence of volunteers [48]. These two processes support each other in a continuous cycle, contributing to disaster resilience.

It is well-known that individuals involved in disaster response processes experience certain psychological effects [49,50,51]. Volunteers in the Kahramanmaraş earthquake reported feelings of helplessness, sadness, fear, anger, and shock while in the disaster areas. These emotions were attributed to the inability to cope with the scale of destruction, both individually and collectively, due to insufficient capacity, as well as the losses witnessed, even among volunteers with high self-efficacy. Furthermore, the preventable nature of the destruction, combined with its failure to be mitigated for various reasons, contributed to feelings of anger. After returning from the disaster areas, participants continued to experience sadness, as well as guilt and hopelessness. These emotions were linked to the destruction and losses observed, sadness for the affected areas, and a sense of helplessness due to the concept that nothing would change despite their anger toward the lack of disaster risk management efforts. Additionally, one of the striking findings was that participants developed a sense of guilt stemming from the disparity between their possessions and the extreme losses experienced by disaster victims, even though they were not responsible for the disaster. These emotions led participants to isolate themselves from society and undergo significant changes in their outlook on life.

The severe destruction caused by disasters can result in significant trauma for both disaster victims and disaster personnel, including volunteers [50,51]. Participants reported experiencing severe psychological impacts after returning from disaster relief efforts, including sleep disturbances, social withdrawal, and a persistent sense of reliving the earthquake. Previous studies have shown that some individuals experience serious psychological issues following severe trauma, such as depression, anxiety, sleep disorders, and PTSD [10].

In contrast to the negative psychological effects stemming from trauma, some individuals exhibit positive changes following traumatic experiences. This phenomenon is closely linked to individuals’ self-efficacy. The levels of PTG among those involved in disaster relief are positively correlated with their self-efficacy [52]. Individuals with high self-efficacy are more likely to experience positive changes after trauma, such as disasters. These changes often result in individuals becoming stronger compared to their pre-trauma state [10].

In this study, it was determined that participants experienced certain positive changes following their experience of the Kahramanmaraş earthquake. These changes were observed in self-concept, life philosophy, and relationships with others. Participants reported gaining a greater awareness of their self-identity and developing a more positive concept of their self-worth. In terms of life philosophy, participants expressed changes such as a stronger connection to life and a detachment from material values. Additionally, it was found that participants, influenced by the loss of life caused by the disaster, placed greater value on individuals in their social circles and strengthened their relationships.

This study has several limitations. One limitation is that the research focuses solely on volunteers involved in the Kahramanmaraş earthquakes, making the findings non-generalizable to other earthquakes. Another limitation is that the study concentrates exclusively on earthquake disasters and does not provide insights into PTG among volunteers in other types of disasters, such as floods or wildfires. Therefore, future research could adopt a broader scope by including volunteers who have participated in various types of disaster relief efforts, leading to more comprehensive findings.

## 5. Conclusions

Disasters can be defined as traumatic events that have a negative impact on society and the psychology of individuals in society in general, but some positive changes can be observed in individuals who are directly or indirectly exposed to the disaster. The experiences of the researchers after their observations in the disaster area for about two months and their dialogues with the volunteer individuals after the field were the driving factors in conducting this study. In this context, it was found that the volunteers mainly showed post-traumatic growth after the disaster. Not only those directly affected by disasters but also individuals who provide indirect assistance can be significantly impacted by these experiences. Factors such as the inability to provide effective help, confronting the reality of death, and exposure to tragic scenes can trigger psychological trauma. However, certain positive changes can also be observed in individuals following trauma. These changes may manifest in improved self-concept, life philosophy, and relationships with others, enabling individuals to respond with greater maturity. This study found that PTG was observed in various dimensions among individuals and that differences in the “CSC” dimension were identified based on gender, earthquake experience, and the institutions where volunteer activities were carried out. Additionally, analyses of semi-structured interviews revealed that the majority of participants expressed a predominance of negative emotions, exhibited trauma symptoms, and reported significant CSC and CPL after returning from the field. Thus, although disasters are generally associated with negative connotations, this study highlights that they can also lead to positive transformations in how some individuals perceive themselves, their lives, and the people around them. It may be useful to conduct post-disaster psychological support activities, group therapies, and other activities that enable the sharing of experiences and emotions among the people who will volunteer in disasters. It may also be useful to conduct seminars and training on the basis of experience gained by experts in their field and individuals who have volunteered in the field, especially for individuals who apply to volunteer in future periods, with insight into possible problems that individuals may face in the future. In addition, it is necessary for the administration to monitor PTSD and PTSD incidents among volunteers sent by the public to work in the region after disasters and to create modules for these phenomena in training and exercises in the preparation phase of disaster risk management. In disaster crisis management, the public administration should follow the volunteers in this regard to develop the volunteer system by considering the long-term psychological effects. In the case of need or demand, public and private stakeholders should enact the necessary activities and arrangements in cooperation within the framework of governance. Although there are deficiencies in disaster plans, legislation, and policies in Türkiye, it is important for the functionality of the disaster management system and disaster resilience to carry out studies on this issue. Based on these findings, it is recommended that future research be conducted with different groups and in countries with diverse cultural contexts to further explore this subject.

## Figures and Tables

**Table 1:** Demographic characteristics of Posttraumatic Growth Inventory participants.

Variable	Groups	Frequency (F)	Percentage (%)
Gender	Male	79	46.7
	Female	90	53.3
Age	18–25 years	95	56.2
	26–35 years	50	29.6
	36–45 years	24	14.2
Education	Below Bachelor’s Degree	24	14.2
	Undergraduate	127	75.1
	Graduate	18	10.7
Marital Status	Married	39	23.1
	Single	130	76.9
Region Of Residence	Province	107	63.3
	District	55	32.5
	Village	7	4.1
Earthquake Experience	Experienced	114	67.5
	Never Experienced	55	32.5
Organization Conducting Volunteering Activities	AFAD	63	37.3
	Red Crescent	13	7.7
	Other Civil Society Organizations	36	21.3
	Under the Roof of the Organization	31	18.3
	By Their Own Means	26	15.4

**Table 2:** PTGI subscale and total scores by participant characteristics.

Features	CSC ^1^ Mean ± SD	CPL ^1^ Mean ± SD	CRO ^1^ Mean ± SD	PTGI TS ^1^ Mean ± SD
Gender				
Male (*n* = 90)	4.22 ± 0.94	3.66 ± 0.99	3.54 ± 1.17	3.90 ± 0.89
Female (*n* = 79)	3.77 ± 1.14	3.22 ± 1.06	3.26 ± 1.30	3.49 ± 1.06
*p* *	<0.006	<0.007	0.148	<0.008
Age				
18–25 (*n* = 95)	4.02 ± 1.13	3.49 ± 1.11	3.43 ± 1.28	3.73 ± 1.05
26–35 (*n* = 50)	4.05 ± 1.00	3.37 ± 0.97	3.38 ± 1.21	3.69 ± 0.94
36 years and older (*n* = 24)	3.67 ± 0.95	3.27 ± 0.97	3.29 ± 1.18	3.46 ± 0.90
*p* **	0.317	0.582	0.874	0.511
Marital Status				
Married (*n* = 39)	4.02 ± 1.00	3.44 ± 1.32	3.77 ± 0.97	3.61 ± 1.01
Single (*n* = 130)	3.97 ± 1.10	3.38 ± 1.22	3.66 ± 1.01	3.37 ± 1.06
*p* *	0.778	0.788	0.548	0.208
Education				
Below Bachelor’s Degree (*n* = 24)	3.61 ± 1.26	3.05 ± 1.07	3.30 ± 1.36	3.37 ± 1.14
Undergraduate (*n* = 127)	4.05 ± 1.04	3.47 ± 1.05	3.40 ± 1.24	3.73 ± 0.98
Graduate (*n* = 18)	3.97 ± 0.94	3.57 ± 0.98	3.48 ± 1.14	3.74 ± 0.90
*p* **	0.183	0.162	0.885	0.272
Region of Residence				
Province (*n* = 107)	4.00 ± 1.02	3.46 ± 1.03	3.53 ± 1.23	3.73 ± 0.97
District (*n* = 55)	3.90 ± 1.22	3.37 ± 1.11	3.13 ± 1.21	3.56 ± 1.08
Village (*n* = 7)	4.24 ± 0.48	3.35 ± 0.85	3.40 ± 1.49	3.78 ± 0.64
*p* **	0.693	0.874	0.146	0.578
Earthquake Experience				
Experienced (*n* = 114)	3.86 ± 1.09	3.31 ± 1.23	3.59 ± 1.04	3.37 ± 1.12
Never experienced (*n* = 55)	4.22 ± 0.99	3.56 ± 1.27	3.86 ± 0.88	3.53 ± 0.89
*p* *	0.046	0.233	0.101	0.334
Organization Conducting Volunteering Activities				
AFAD (*n* = 63)	4.19 ± 1.05	3.58 ± 1.04	3.52 ± 1.31	3.86 ± 1.01
Red Crescent (*n* = 13)	3.78 ± 1.22	3.32 ± 1.21	3.60 ± 1.28	3.60 ± 1.20
Other Civil Society Organizations (*n* = 36)	3.96 ± 1.02	3.39 ± 1.10	3.61 ± 1.22	3.71 ± 0.99
Under the Roof of the Organization (*n* = 31)	3.50 ± 1.25	3.08 ± 1.00	2.79 ± 1.20	3.21 ± 1.04
By Their Own Means (n = 26)	4.16 ± 0.69	3.55 ± 0.93	3.41 ± 0.95	3.80 ± 0.77
*p* **	0.041	0.260	0.050	0.051

^1^ Change in Self-Concept (CSC), Change in Philosophy of Life (CPL), and Change in Relationships with Others (CRO). PTGI TS: Posttraumatic Growth Inventory Total Score. Mean ± Standard Deviation * *T*-Test ** ANOVA.

**Table 3:** Demographic characteristics of the interview participants.

ID	Age	Gender	Profession	Educational Status	Marital Status	Earthquake Experience Status	Interview Time *
P1	28	Female	Research Assistant	Postgraduate	Single	Yes	33 min 35 s
P2	27	Male	Lecturer	Postgraduate	Single	Yes	33 min 53 s
P3	41	Male	OHS Specialist	Bachelor’s degree	Married	No	31 min 33 s
P4	24	Female	Self-Employed	Bachelor’s degree	Single	Yes	22 min 43 s
P5	26	Male	Firefighter	Postgraduate	Single	Yes	27 min 44 s
P6	32	Male	EMT	Postgraduate	Single	Yes	30 min 02 s
P7	21	Female	Student	High School	Single	Yes	25 min 12 s
P8	32	Female	Lecturer	Postgraduate	Single	Yes	59 min 59 s
P9	23	Male	Search and Rescue Volunteer	High School	Single	Yes	46 min 53 s
P10	32	Female	Lecturer	Postgraduate	Single	Yes	22 min 33 s
P11	31	Female	Nurse	Bachelor’s degree	Single	No	27 min 27 s
P12	32	Female	Assistant Prof.	Postgraduate	Married	Yes	25 min 12 s
P13	38	Female	Security	Bachelor’s degree	Single	No	21 min 05 s
P14	25	Female	Unemployed	Bachelor’s degree	Single	No	18 min 57 s

* min: minutes.

**Table 4:** Participants’ levels of PTG across dimensions based on the PTG scale.

Subdimensions of PTG	A.M ± S.D.	Med (IQR)	Min–Max
CSC	3.98 ± 1.07	4.20 (4.75–3.30)	1.00–6.00
CPL	3.42 ± 1.05	3.66 (4.16–2.66)	1.00–5.67
CRO	3.39 ± 1.24	3.40 (4.40–2.50)	1.00–6.00
PTGI Total Score	3.68 ± 1.00	3.80 (4.47–3.09)	1.00–5.90

**Table 5:** Summary information for qualitative and quantitative sections.

Theme/PTGI Subdimension	Description/Content	Representative Codes/Indicators	Number of Coding	Total Number of Participants (*n* = 14)	Participant Code
Change in Self Perception	Change in self-perception of the individual after trauma	“*I thought I should love myself more and make myself happy*” (P1) “*I don’t make impulsive outbursts as much as I used to. I started to empathize*” (P3) “*I discovered my capacity*” (P4)	20	10	P1, P3, P7, P8, P9, P10, P11, P12, P13
Change in Philosophy of Life	Transformation in thinking about the meaning of life and priorities	“*the goods, property, everything in the world is empty. So you think that only my health and my life are important and so on*” (P1) “*Questioning that life is really empty and that we live in a two-day world*” (P10) “*I have now seen more clearly that the most valuable asset in life is time, health and well-being*” (P12)	15	10	P1, P2, P3, P4, P5, P7, P8, P9, P10, P12
Change in Relationships with Others	Positive transformations in the individual’s social relationships	“*I used to be a person who intervened in events. I have become more of an observer. I have become a person who watches my social life, the people around me or my family from a distance*” (P5) “*My family ties are normally strong, but they became stronger after the earthquake*” (P7) “*Our social sharing has increased even more*” (P8)	6	5	P4, P5, P7, P8, P12

Theme/PTGI Subdimension

Description/Content

Representative Codes/Indicators

Number of Coding

Total Number of Participants (*n* = 14)

Participant Code

## Data Availability

The data presented in this study are available upon request from the corresponding author due to (cannot be shared directly within the scope of the Personal Data Protection Law).
